# Construction of deep learning-based disease detection model in plants

**DOI:** 10.1038/s41598-023-34549-2

**Published:** 2023-05-05

**Authors:** Minah Jung, Jong Seob Song, Ah-Young Shin, Beomjo Choi, Sangjin Go, Suk-Yoon Kwon, Juhan Park, Sung Goo Park, Yong-Min Kim

**Affiliations:** 1 Department of Functional Genomics, KRIBB School of Biological Science, Korea University of Science and Technology (UST), Daejeon, Republic of Korea; 2 Euclidsoft Co., Ltd, Daejeon, Republic of Korea; 3 Plant Systems Research Center, Korea Research Institute of Bioscience and Biotechnology (KRIBB), Daejeon, Republic of Korea; 4 Department of Bioinformatics, KRIBB School of Bioscience, Korea University of Science and Technology (UST), Daejeon, Republic of Korea; 5 Department of Environmental Horticulture, University of Seoul, Seoul, Republic of Korea; 6 Biosystems and Bioengineering Program, KRIBB School of Bioscience, Korea University of Science and Technology (UST), Daejeon, Korea; 7 Disease Target Structure Research Center, Korea Research Institute of Bioscience and Biotechnology (KRIBB), Daejeon, Republic of Korea; 8 Digital Bioinnovation Center, Korea Research Institute of Bioscience and Biotechnology (KRIBB), Daejeon, Republic of Korea

**Keywords:** Classification and taxonomy, Image processing, Machine learning

## Abstract

Accurately detecting disease occurrences of crops in early stage is essential for quality and yield of crops through the decision of an appropriate treatments. However, detection of disease needs specialized knowledge and long-term experiences in plant pathology. Thus, an automated system for disease detecting in crops will play an important role in agriculture by constructing early detection system of disease. To develop this system, construction of a stepwise disease detection model using images of diseased-healthy plant pairs and a CNN algorithm consisting of five pre-trained models. The disease detection model consists of three step classification models, crop classification, disease detection, and disease classification. The ‘unknown’ is added into categories to generalize the model for wide application. In the validation test, the disease detection model classified crops and disease types with high accuracy (97.09%). The low accuracy of non-model crops was improved by adding these crops to the training dataset implicating expendability of the model. Our model has the potential to apply to smart farming of Solanaceae crops and will be widely used by adding more various crops as training dataset.

## Introduction

Crop disease management is important in agriculture to increase yield and quality by reducing the economic and aesthetic damage caused by plant diseases. Although research into the causes and effective treatments for crop diseases is actively underway, monitoring plant health and early detection of pathogens are critical to reduce disease spread and facilitate effective management^1^. Detecting and protecting crops from pathogens is labor-intensive and time-consuming, making it virtually impossible for humans to analyze each plant^2^. Therefore, research on combining and applying new technologies to efficiently detect diseases has been conducted, and recently, research on detecting plant diseases in leaves using artificial intelligence (AI) is in progress^3^. Continued development of improved classification models, such as disease detection, or plant health monitoring, may enable AI-supported decision-making systems for smart agriculture^4^. Various studies have been carried out to apply deep learning algorithms more precisely to disease detection, such as applying newly developed architectures^5,6^, automatically detecting and classifying lesions in plant images^7^, or conducting research on preprocessing methods for incomplete images^8^ for practical use.

Artificial Neural Network (ANN) is an AI technology with an analytic system inspired by the nerve system of the human brain that mimics the way the brain processes information^9^. ANN contains a three component processing unit consisting of input, hidden, and output layers^10^. Nodes of individual layers are connected to nodes of adjacent layers. Convolutional Neural Network (CNN) is a specialized method to recognize or assign images and consists of fully connected layers, numerous convolution layers, and pooling layers^11^. Three types of layers are arranged and connected differently depending on the model architectures and model performance is affected by this architecture^12^. AlexNet^13^, VGG19^14^, GoogLeNet^15^, ResNet^16^, and EfficientNet^17^ are pre-trained CNN models created by changing the number, composition, arrangement, or calculation method of three types of layers, and ranked high in competition, the ImageNet Large Scale Visual Recognition Challenge (ILSVRC)^18^. These are pre-trained CNN models whose performance has been confirmed. CNN architecture development has focused on improving accuracy or efficiency. However, each architecture has its own unique characteristics and appropriate architectures are required for individual datasets^19,20^. Recently, these CNN algorithms have been used to develop various tools or programs for the detection or assignment of objectives in various fields^21^. In particular, detecting disease of plants using CNN algorithms were preferred rather than other deep learning algorithms^22^.

Recently, CNN-based research have been carried out in plants^23^ and CNN analysis showed high performance in phenotypic analysis^24^. Thus, CNN-based phenotypic analyses have been reported in various crops such as classification of plant species, and detection of plant diseases in single or multiple species. To classify 44 species of the ILSVRC2012 dataset were classified using a pre-trained model^18^ proposed by ImageNet, and accuracy was shown as 97.7%^25^. In addition, about 94 percent accuracy was shown from VGG and AlexNet analysis with 42 species of image data from the IHLD dataset using Flavia^26,27^. Further analysis with disease data, 21 classes of 5 species were classified with MobileNetv2 with an accuracy of 90.38%^28^. Other study for classification of diseases for Bell pepper, an accuracy of 99.75% was shown using LBP + VGG-16 fused features^29^, Potato leaf were used for classification of diseases using a VGG16 and VGG19 architecture, and accuracy was shown 91%^30^. In tomato, ResNet50 showed a 97% accuracy in classification of six prevalent diseases in tomato leaf^31^.

Here, a disease detection model for plants was constructed using images of diseased-healthy plant pairs and AI algorithms. To develop the disease detection model, 24,101 image pairs from nine crops were used with a CNN algorithm consisting of five pre-trained models. The detection model was consistent with three step classification models. When constructing the detection model, the most accurate pre-trained model was selected as the final classification model from five different pre-trained CNN models. In addition to disease detection, the applicability to smart agriculture was confirmed by performing verification on crops not used in disease detection model training. Additionally, to investigate the pattern of symptoms and lesions of disease using our disease detection model, images of crop disease lesions were tested.

This study has the following key contributions: First, a stepwise disease detection model was developed, and each step had specific purpose. To improve the accuracy, an optimal pre-trained CNN model was selected for each step. Second, the stepwise model was able to provide flexibility and efficiency to users by selecting individual steps according to their purpose. Efficient analysis might be possible when this model will be applied to the industrial field. Third, various types of validation tests were carried out to apply the industrial filed. To confirm the ability to disease detection, validation tests were carried out using lesion or whole leaf. To investigate effect of training data quality, and processed data and filed data were also tested. Then, to provide an ‘unknown’ detecting function, 'unknown' was defined to increase accuracy.

## Results

### Crop detection and classification using leaf images

**Figure 1:**
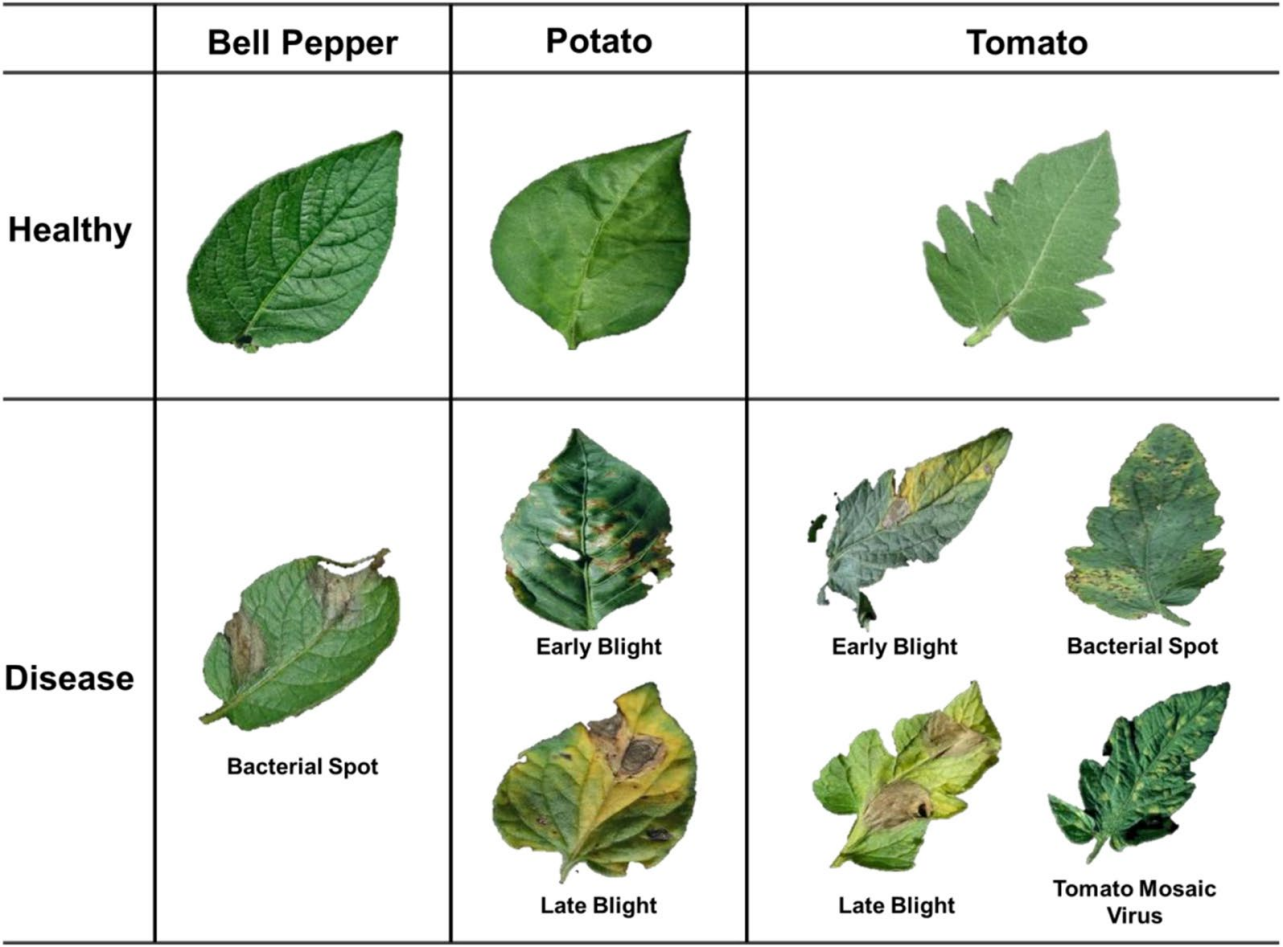
Overview of the dataset for disease detection models. Examples of image data from Solanaceae including bell pepper, potato, and tomato.

**Figure 2:**
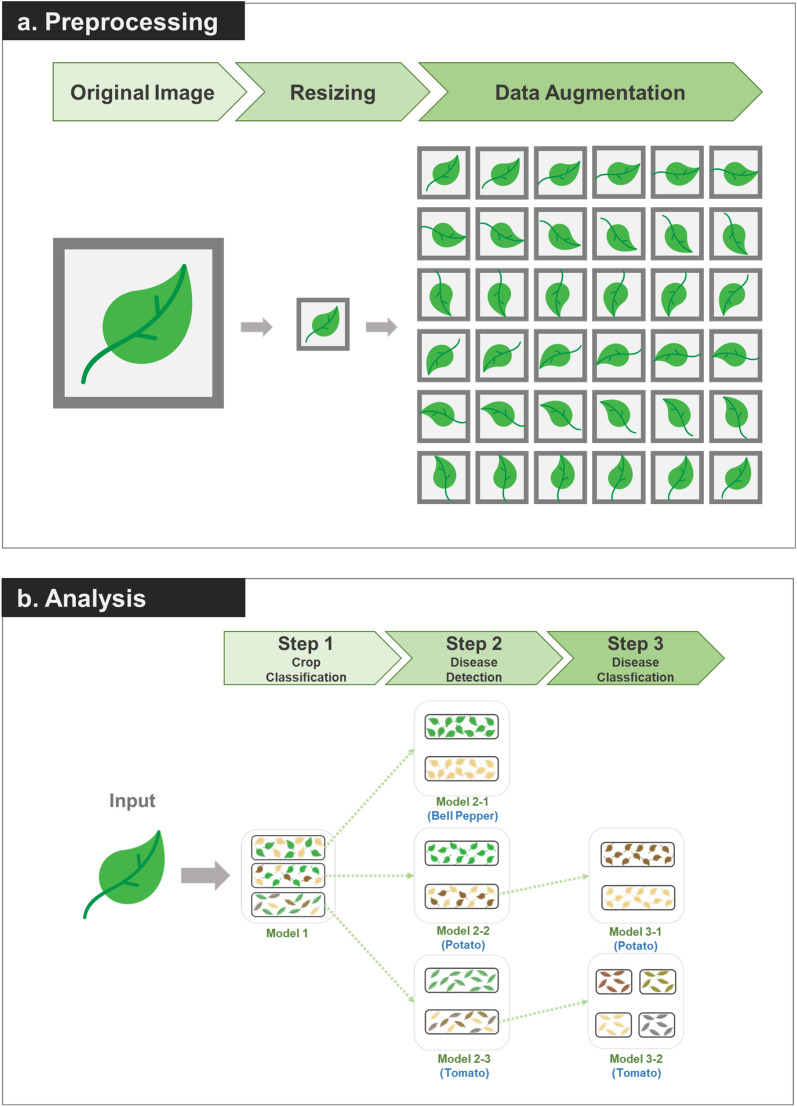
Workflow of the disease detection model. (**a**) Phenotypic data preprocessing after resizing the original image data and data augmentation through rotation. (**b**) Stepwise disease detection model of Solanaceae through a deep-learning algorithm.

A three step detection model for plant diseases was constructed using healthy and disease leaf images of bell pepper, potato and tomato (Fig. 1). In total, six submodels based on CNN were developed for each step of the classification model after data augmentation (Fig. 2). Five different pre-trained models such as ResNet50, AlexNet, GoogLeNet, VGG19, and EfficientNet were tested for each crop to develop the model (Supplementary Table S1). Each pre-trained model was optimized by tuning hyperparameters, such as batch size, epoch size, optimizer, activation function, learning rate, early stopping function, and loss function (Supplementary Table S2).

In step 1, the crop classification model was constructed using diseased and healthy leaf images of 1911 bell pepper, 1448 potato, and 3150 tomato. Species of crops were recognized by submodel and assigned to one of three categories, bell pepper, potato, or tomato. After model training and validation, of the five different pre-trained CNN models, the model showing the highest accuracy using the separated test set was selected as the final model for step 1. As a result, it showed high accuracy in the order of EfficientNet, GoogLeNet, VGG19, AlexNet, and ResNet50 (Table 1). The classification model of EfficientNet architecture showed the highest accuracy of 99.33% and was selected as the final model for further analysis (Table 1). In addition to accuracy, measurement methods of were applied to measure the performance of the classification model. The precision, recall, and F1-score were also shown as high rate (Supplementary Table S3).

**Table 1:** Result of validation and test using five pre-trained CNN models.

Step		Crop	Pre-trained model	Accuracy	
				Validation	Test
I	Crop classification	All	ResNet50	92.34%	91.84%
			AlexNet	97.62%	96.87%
			GoogleNet	98.16%	99.08%
			VGG19	96.86%	98.71%
			EfficientNet	98.54%	99.33%
II	Disease detection	Bell Pepper	ResNet50	100.00%	98.32%
			AlexNet	98.96%	99.16%
			GoogleNet	100.00%	100.00%
			VGG19	99.74%	99.58%
			EfficientNet	99.48%	99.58%
		Potato	ResNet50	100.00%	99.45%
			AlexNet	99.31%	98.90%
			GoogleNet	99.66%	99.45%
			VGG19	100.00%	100.00%
			EfficientNet	100.00%	99.45%
		Tomato	ResNet50	99.68%	99.75%
			AlexNet	99.53%	99.45%
			GoogleNet	99.62%	99.62%
			VGG19	99.12%	99.62%
			EfficientNet	99.62%	98.23%
III	Disease classification	Potato	ResNet50	99.25%	98.80%
			AlexNet	98.88%	99.40%
			GoogleNet	99.63%	99.40%
			VGG19	99.63%	99.40%
			EfficientNet	97.75%	99.40%
		Tomato	ResNet50	92.92%	87.80%
			AlexNet	92.47%	95.45%
			GoogleNet	94.29%	95.81%
			VGG19	95.66%	95.08%
			EfficientNet	96.35%	97.09%

### Disease detection for individual crops

**Figure 3:**
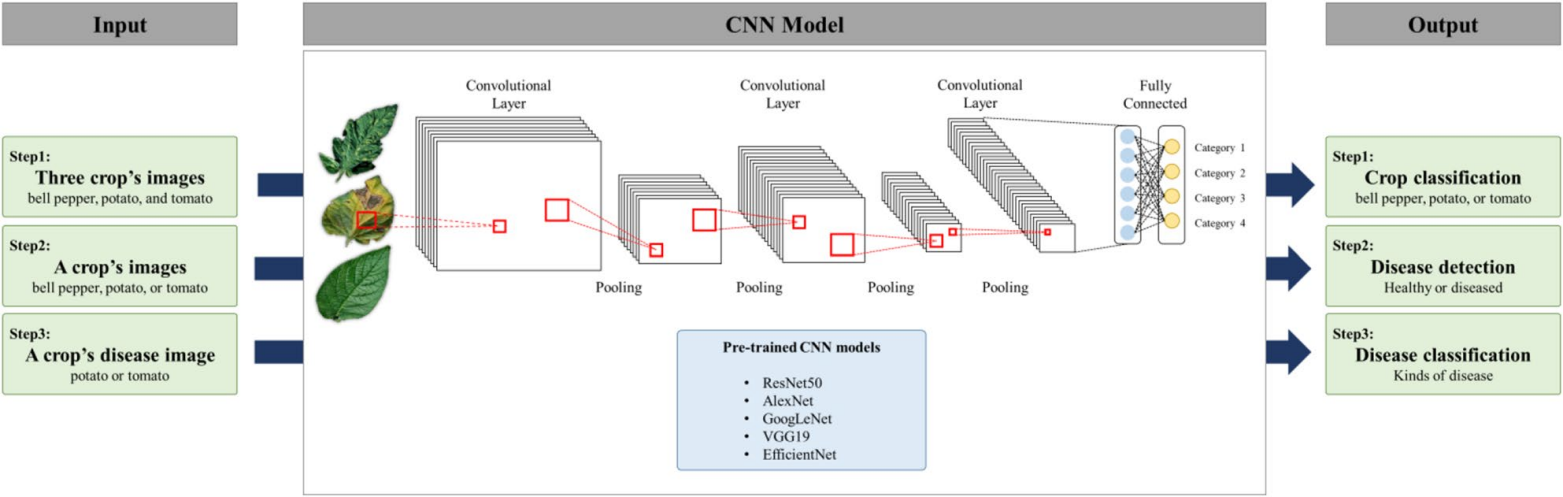
Diagram of disease detection model using CNN model.

After accurate crop recognition, detection models were used to determine disease occurrence for individual crops by detecting the presence or absence of disease symptoms or patterns of symptoms in step 2 (Fig. 3). Three individual models were developed to detect disease for bell pepper, potato, and tomato. As the bell pepper disease detection model, 1,165 healthy images and 746 diseased images were used for model training and validation, and GoogLeNet showed the highest accuracy in test. The accuracy of EfficientNet, VGG19, AlexNet, and ResNet50 were followed (Table 1). GoogLeNet, which showed the highest accuracy, was selected as the final model. In test, it showed an accuracy of 100.00%, and precision, recall, and F1-score were all 100.00% (Supplementary Table S3).

For potato disease detection model used 118 healthy images and 1,330 diseased images for model training and validation, and VGG19 showed the highest accuracy in test. The accuracy of EfficientNet, GoogLeNet, ResNet50, and AlexNet were follows. The tests performed on the VGG19 showed the highest accuracy with 100.00%, and precision, recall, and F1-score were all 100.00% (Supplementary Table S3).

As for the tomato disease detection model, 974 healthy images and 2,176 diseased images were used, and ResNet50 showed the highest accuracy in test. The other four models, GoogLeNet, VGG19, AlexNet, and EfficientNet showed less than the final model. The test performed on the ResNet50 showed the highest accuracy with 99.75%, and precision, recall, and F1-score were 99.75%, 99.75%, and 99.75%, respectively (Supplementary Table S3).

### Classification of diseases on leaves

If a disease was detected in step 2, it was necessary to determine the type of disease. In step 3, a disease classification model was created for potato and tomato (Supplementary Table S4). Two or more types of disease data were obtained for both potato and tomato. For potato, disease data (1662 images) was divided into two diseases: early blight (929 images) and late blight (733 images). For tomato, disease data (2,177 images) was divided into four diseases: bacterial spot (1,670 images), early blight (302 images), late blight (467 images), and tomato mosaic virus (281 images). To distinguish the two potato diseases, five different models were created using pre-trained CNN models. The highest test accuracy was shown in order of VGG19, EfficientNet, AlexNet, and GoogLeNet and ResNet50 (98.80%) was shown slightly low accuracy (Table 1). The final model test results showed that the accuracy, precision, recall, and F1-score were 99.40%, 100.00%, 98.64%, and 99.32%, respectively (Supplementary Table S3).

In the case of tomato, models were created and tested to distinguish the four diseases. EfficientNet had the highest test accuracy with 97.09%, and GoogLeNet, AlexNet, VGG19 showed accuracy of over 95% (Table 1). However, ResNet50 (87.80%) showed relatively low accuracy compared to the other four models. As the result of the final model, precision, recall, and F1-score were 97.19%, 97.09%, and 97.12%, respectively (Supplementary Table S3). The result of both disease classification model tests confirmed the test set data were classified with high accuracy (> 97%). Because other performance measures, such as precision, recall, and F1-score, also showed high scores, the models were able to classify early and late blight, without bias. These results confirmed the stability of the classification model.

### Validation of the classification model using other crops

**Figure 4:**
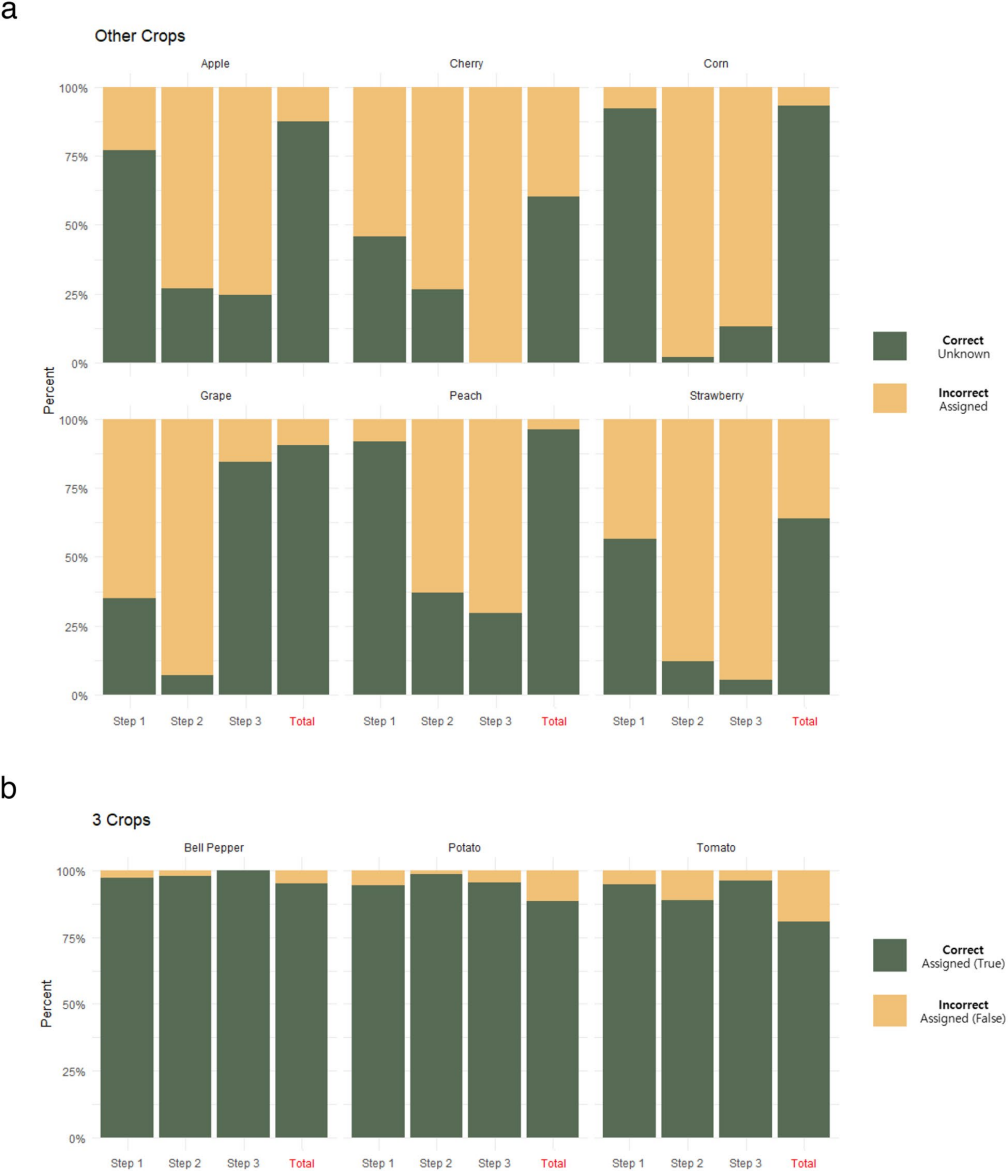
Evaluation of the results of the stepwise detection model for plant diseases. (**a**) Correct evaluation results for each step using six other crops, which were not used to construct the model. (**b**) Correct evaluation results for each step using the three crops used to construct the model.

To generalize developed the classification model to other field such as model for classification of plant species or determining medicinal plants as well as smart farming, validation of other crops not used for model construction is required. To that end, the classification model was validated using leaf images of six crops: apple, cherry, corn, grape, peach, and strawberry. Since crops not used in model construction could not be identified using the disease detection model, these crops (non-model crops) were correctly classified as ‘unknown’. In case of corn and peach data, 92.06% and 91.66%, respectively, were classified as ‘unknown’, showing high accuracy in step 1. Apple (76.92%), strawberry (56.72%), cherry (45.67%), and grape (34.94%) data were less accurate. Apple in step 2 and grape in step 3 were classified as 'unknown' with 87.30% and 90.64% frequency, respectively. Cherry and strawberry were classified as 'unknown' less frequently, 60.07% and 64.04%, respectively (Fig. 4a and Supplementary Table S5). Large differences were found in evaluating performance according to crop characteristics with non-model crops, whereas model crops were correctly classified in each step (Fig. 4b and Supplementary Table S6).

Because the evaluation of strawberry data showed relatively low accuracy, an additional model (model 1 with strawberry) that included strawberry was constructed to compare with the previously established step 1 model (model 1 without strawberry) (Supplementary Fig. S1a). Further validation was performed using diseased strawberry images taken in the field from 'The Open AI Dataset Project (AI-hub (https://aihub.or.kr/aidata/30729), Republic of Korea)' (Supplementary Fig. S1b). The accuracy of the strawberry detection was 56.72% in the model 1 without strawberry and 96.94% in the model 1 with strawberry (Supplementary Fig. S1c). The accuracy was increased by adding strawberry data during model construction by more than 30%. The results of validation using data from field indicated that 63.64% (70 of 110) of images were classified correctly in the model 1 without strawberry and 74.55% of (82 of 110) images were classified correctly in the model 1 with strawberry (Supplementary Fig. S1c). Of the 110 AI-hub data images, 58 images were correctly classified in both the model 1 without strawberry and the model 1 with strawberry, and 12 and 24 images, respectively, had different results depending on the classification model. Taken together, these results indicate that the model 1 with strawberry was able to classify strawberry more accurately.

### Evaluation of the classification model using lesion cropped images

**Figure 5:**
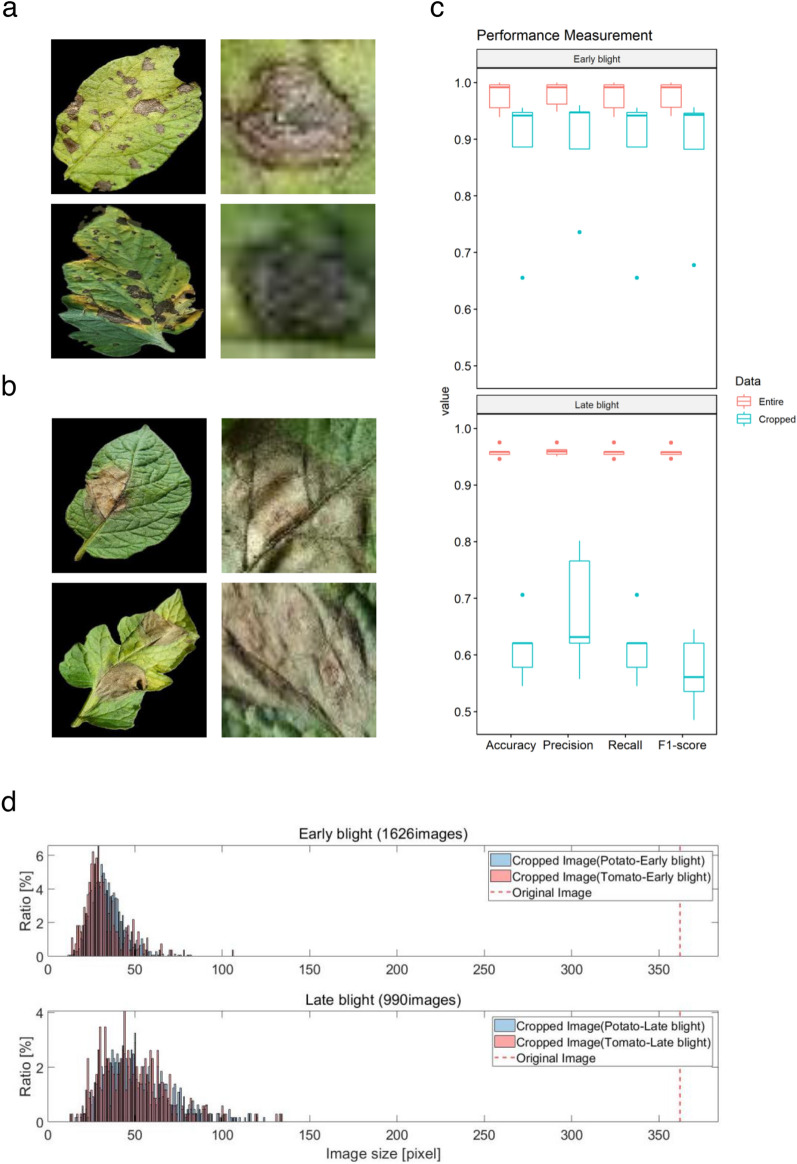
The effect of image resolution in construction of the classification model. Entire or cropped lesion images of (**a**) early blight and (**b**) late blight images. Potato and tomato leaf images are shown in the upper and lower panel, respectively. (**c**) Detection performance from the cross validation set, the upper plot is for early blight and the other one is for late blight. (**d**) Distribution of diagonal resolution for cropped lesion images was shown with the original image’s resolution (red line).

Evaluation of the classification models was conducted using entire leaf images and lesion cropped images to investigate the effect of apparent symptoms on the model (Fig. 5). First, crop classification models were created using entire leaf and lesion cropped images of early blight and late blight disease present in both potato and tomato (Fig. 5a,b). All test sets of entire leaf images were correctly classified. The highest accuracy was shown in GoogLeNet pre-trained model, which classified early blight with 100.00% accuracy. For late blight, the accuracy was 97.51% (Table 2 and Fig. 5c). The accuracy of the classification models was reduced when analyzing lesion cropped images in both early blight and late blight (Table 2 and Fig. 5c). GoogLeNet pre-trained model had the highest accuracy predicting early blight, which was 95.56%. For late blight, VGG19 pre-trained model showed the highest accuracy with 70.62%. The accuracy was significantly different between early blight and late blight, especially when using lesion cropped images (p = 0.021, Wilcoxon rank sum test). Higher accuracy was shown in predicting early blight in both entire leaf and lesion cropped images. In addition, the prediction performance of the classification model using lesion cropped images were lower than that of entire leaf images. These results might be caused by the low resolution (20 × 20 pixels) of lesion cropped images. Therefore, the effect of resolution was investigated in both early blight and late blight (Fig. 5d), which indicated that image resolution did not affect the performance of classification models in either disease. One of the major differences between both diseased images was the features of the symptoms (Fig. 5a,b). For early blight, the color of the lesion is dark and clear, whereas the boundary of the lesion is vague and spread with lighter color in late blight (Supplementary Table S4). These data suggest the classification model using CNN may reliably recognize the different symptoms between early blight and late blight.

**Table 2:** Performance measurement of the model for validation using cross-species.

Validation	Disease	Pre-trained model	Performance			
			Accuracy	Precision	Recall	F1-score
Crop cross	Early blight	GoogLeNet	100.00%	100.00%	100.00%	100.00%
	Late blight	GoogLeNet	97.51%	97.55%	97.51%	97.50%
Cropped image	Early blight	GoogLeNet	95.56%	95.99%	95.56%	95.65%
	Late blight	VGG19	70.62%	80.15%	70.62%	64.53%

## Discussion

Crop monitoring, especially smart farming, plays an important role in agriculture to increase crop yield or quality. Smart farming has become a crucial factor that has added indispensable value to agriculture and can help maintain high quality crops. Thus, development of AI-based disease monitoring programs will be an essential application of basic science to agriculture. In present study, a deep learning-based disease detection model was constructed to mimic disease recognition by humans in three major crops (Fig. 2). Although many studies have been conducted to classify diseases of bell pepper, potato, and tomato using CNN models^32–35^, most of them a disease of a specific crop as one category and classify them all at once. However, our model differs in analyzing several stages because it assumes actual agricultural use. It can sequentially provide information about 'which crop' is ‘diseased’ and ‘what the disease is’. The disease detection model was designed to recognize crops, detect disease, and determine the type of disease (Fig. 2).

Although the high-accuracy pre-trained CNN model was different for each step, validation using model crops indicated that the accuracies of the classification models were high enough for use in agriculture. In step 1, EfficientNet (99.30%) showed the highest accuracy among five pre-trained CNN models (Table 1). The model with the highest accuracy in step 2 was different for each crop. In step 3, EfficientNet was shown the highest accuracies in potato (99.40%) and tomato (97.09%). For tomato disease classification, ResNet50 has the highest accuracy (99.75%) in step 2, but is more than 5% less accurate than other pre-trained CNN models in step 1 (91.84%) and step 3 (87.80%). In step 1, bell pepper and potato are similar, and in step 3, the bacterial spot and early blight at tomato symptoms are similar, so the accuracy seems to be low. Collectively, ResNet50 seems to be less accurate when classifying images with high similarity, which is the same as the results of the previous study. According to previous studies, the CNN algorithm is considered suitable for disease detection of plants compared to others^22^. Among CNN pre-trained models, EfficientNet is shown the highest accuracy in recent studies^36–38^. In this study, EfficientNet showed good detection performance in several plant phenotypic data. However, detection performances depended on the morphological features or disease symptoms and the best performance might be shown in other pre-trained models. Thus, multiple pre-trained models need to be tested during the training step and determined optimal pre-model based on the accuracy of disease detection from evaluation data.

Especially, to analyze data not used in the model, an ‘unknown’ category was defined and a test was conducted. To generalize the detection model, non-model crops must be distinguished and assigned them to the ‘unknown’ category. The classification model was further evaluated using six non-model crops, revealing relatively low accuracies compared to model crop (Fig. 4a). The 'unknown' was defined as the probability of correct assignment to its own category. Corn and peach, which show phenotypic differences in leaf shape, were mainly assigned to the ‘unknown’ category in step 1, and most were filtered out during step 1 (crop classification). Due to a similar phenotype, apple was classified as potato in step 1, and most of the misassigned images were classified as 'unknown' in step 2 (disease detection). Apple leaf lesions have a specific characteristic that is distinguished from the model construction data. Similarly, most grape images were classified as tomato in steps 1 and 2, due to similar phenotype, but were classified as 'unknown' in step 3. These results might be due to crop-specific lesion phenotypes of grapes. Cherry and strawberry, which have round-shaped leaves, were frequently misclassified as bell pepper and potato, respectively. However, these lower accuracies were improved by adding non-model crops to training datasets for model construction (Supplementary Fig. S1). In addition, classification models constructed with high quality preprocessed image data showed relatively higher performance compared to classification models constructed using field data (Supplementary Fig. S1c). Collectively, these data suggest the accuracy of the classification model depended on the depth, quality, and variety of image data for training during model construction.

To further investigate the accuracy of disease detection, the ability of the classification model to accurately detect the same disease from different crops was examined (Fig. 5). The results indicated that the accuracy of predicting early blight was higher than that of late blight. These differences might be caused by patterns or features of symptoms in diseased leaves that differ between early blight and late blight (Fig. 5a,b). To confirm this hypothesis, lesions of diseased leaves were cropped, and a classification model was constructed using the cropped images of these lesions. These data indicated that the accuracy of models developed using lesion cropped images were decreased in both diseases compared to models developed using an entire leaf and significantly decreased in early blight (Fig. 5c). It is possible that use of low resolution images (20 × 20 pixels) might affect the accuracy of classification model. To address this, a correlation analysis between accuracy and image resolution was carried out (Fig. 5d). In spite of the low resolution images, the crop lesion characteristics appear to be an important factor for classification as they are classified with more than 70.62% accuracy.

To construct a generalized disease detection model, various situations in various field such as management system in smart farming and phenotypic analysis of disease had to be assumed and tested. Various data such as non-model crop images, crop images in field, and cropped images were used for the construction of the model. Especially, the 'unknown' category was newly defined when the probability is less than a predetermined threshold based on the classification probability. The disease detection model can be generalized by unknown category based on this classification probability. Thus, the disease detection model can be applicable to phenotypic research such as verification of seed purity, detection disordered crops or improved crop varieties.

CNN analysis is widely used for classifying or detecting objects using their images. In the field of plant research and agriculture, major phenotypic analysis studies mainly focus on classifying species, detecting tissues or organs, and detecting diseases or stress responses. Thus, CNN analysis can be widely applied to plant phenotypic analysis or smart farming to monitor crops. In agriculture, CNN-based classification analysis is actively used in commercial crops, such as tomatoes^39^, corn^40^, and bananas^41^. These smart farming studies will help to automatically collect data, recognize events, and post-processing that could replace manpower in the near future. Similar to the human disease recognition process, plant disease was detected with sequential steps. This classification model provides a chance to identify crop species, disease occurrence, and disease type using a single image.

A deep learning-based stepwise disease detection model was built and evaluated under various conditions for actual applications. A new category, ‘unknown’, was defined according to the probability of classification, and a cropped image or a field image was also used. But above all, to make the classification model more elaborate, more high quality images of various crops will be required for model construction, as well as an approach for developing a platform to apply this model to applications such as smart farming and studies of plant pathology.

## Conclusion

In current study, a deep learning model was developed to detect multiple crop diseases. The disease detection model is composed of three steps to recognize crops (step 1), to determine disease occurrence (step 2), and to determine disease types (step 3). In each step, the optimal pre-trained CNN model that could be suitable for the data and purpose was selected and configured. To apply this model to the industrial filed, determination of unknown crops using images of crops that have not been used in model construction were added and 'unknown' was defined to prevent false positives. Further studies should add a variety data of crops with and without diseases to create a generally usable disease classification model.

## Materials and methods

### Dataset description

Diseased and healthy plant leaves for the study were obtained from an open-source database, PlantVillage^42^. A total of 8121 healthy leaf images of bell pepper, potato, and tomato were obtained from nine crops. For the detection of disease, a total of 31,061 diseased leaf images were obtained, including bacterial spot of bell pepper, early blight caused by *Alternaria tomatophila* and *Alternaria solani*, and late blight caused by *Phytophthora infestans* of potato or tomato, and bacterial spot, and tomato mosaic virus of tomato (Supplementary Table S7). In the case of tomato, four diseases out of nine diseases were selected. It was selected to accurately detect diseases common to other crops and to identify diseases that threaten farms. Before constructing the disease detection model, low quality images were filtered by horticultural experta (Supplementary Fig. S2). After image filtering, 18,445 and 6481 images of diseased and healthy leaves remained for subsequent analysis. To elaborate stepwise detection model for plant disease was constructed using Solanaceae corresponding to bell pepper, potato, and tomato of nine crops. Removing background of the image was selected instead of annotation using polygon for training set to develop models using shape of leaf or lesion of images (Fig. 1 and Supplementary Table S4).

### Data preprocessing

To construct and test the stepwise detection model, the test data were separated from the entire dataset prior to model construction, to evaluate the performance of the final model. The whole dataset was divided 80:20 using stratified random sampling, with 80% of dataset serving as a training set and the remaining 20% of dataset used as a test set. Model construction and validation were performed using the training set. Five pre-trained CNN models were constructed and trained by sampling 80% of the training set and performing validation using the remaining 20% of the training set. To improve accuracy and reduce time consumption, hyperparameters including batch size, epoch size, optimizer, activation function, learning rate, early stopping function, and loss function were tuned^43^. After that, of the five pre-trained CNN models, the model with the highest accuracy was selected from each pre-trained model. If the accuracy of the models were same, the model with the least loss is selected. Subsequently, the model with the highest test accuracy among the five pre-trained models was selected as the final model.

To improve the performance of the disease detection model, it was necessary to use more data. Therefore, data augmentation was carried out to obtain more image data by manipulating the existing training dataset. The collected data has a large image size and leaf midrib directions are all different, images were resizing original images to 224 pixels and rotated to construct a classification model regardless of the angle of the picture and generate a more elaborate model (Fig. 2a). Brightness or color shifts were not chosen as data augmentation methods because they likely interfere with the features of crop or diseased lesions. In the case of step 1, because all data were used, the amount was considerable. Therefore, the image data was rotated 20 degrees and amplified 18 times to be used as analysis data for computing power. For step 2 and 3, each image was rotated 10 degrees to augment the data 36 times.

### Stepwise detection model for plant diseases

The stepwise detection model was constructed with consecutive submodels to determine crops (step 1), disease occurrence (step 2), and disease types (step 3) by mimicking human detection (Fig. 2b). This model was constructed using the CNN analysis method optimized for image analysis among deep learning analysis techniques (Algorithm 1). Each submodel was developed by fine-tuning using five different pre-trained CNN models: AlexNet, ResNet50, GoogLeNet, and VGG19 which ranked first or second in ILSVRC^44,45^, and EfficientNet which showed good performance in plant classification^36–38^. In step 1, a model to determine and classify cultivars was developed using images of an entire leaf, regardless of presence or absence of disease, in three Solanaceae family crops using five pre-trained CNN models (Fig. 3). In step 2, disease occurrence was determined by dividing healthy and diseased leaves into two groups by submodels. In step 3, disease types were determined by classification models for individual crop diseases using images of diseased leaves classified from step 2. For the bell pepper, only images from a single type of disease were obtained and the classification model could not be developed to distinguish multiple intraspecies diseases (Fig. 2b).
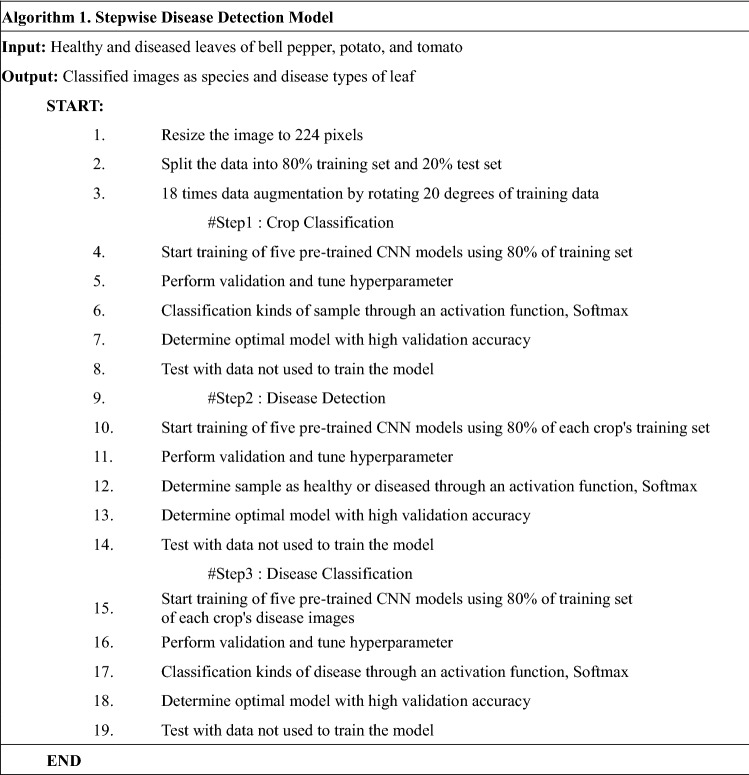


### Evaluation stepwise detection model for plant diseases using diseased image data from other crops

To develop the detection model to a level that can be used for smart farming, apple, cherry, corn, grape, peach, and strawberry were used for evaluation. The six crops that were not used for model construction were defined as 'unknown' because it was impossible to discriminate with our model. Therefore, the probability when determining one of the three crops, bell pepper, potato, and tomato is lower than a threshold value, it is predicted as 'unknown'. The prediction of the 'unknown' is determined in step 1, step 2, and step 3. First, if 'unknown' is determined in step 1, the analysis is finished. Even if it is incorrectly determined that it is not 'unknown' in step 1, it can be determined as 'unknown' through step 2 or step 3. The indicate function was used to classify 'unknown' for each step. The indicate function determines 'unknown' or the crops, 'unknown' or disease existence, and 'unknown' or disease types based on the probabilities of the disease detection model. The indicate function is used separately as ${I}_{1}$, ${I}_{2}$, and ${I}_{3}$ in each step of step1, step2, and step 3. First, indicator function ${I}_{1}$ of step1, for non-model crop evaluation sample $l$ ($l=1, \cdots , L, L = the\,total\,number\,of\,evaluation\,samples)$,

$${I}_{1l}= \left\{\begin{array}{c}unknown\,\,\,\,\,\,\,\,\,\,if\,{p}_{l} < T{h}_{1}\\ \widehat{{c}_{1}}\,\,\,\,\,\,\,\,\,\,\,\,\,\,\,\,\,\,\,\,\,\,\,\,\,\,\,\,\,if\,{p}_{l} \ge T{h}_{1},\end{array}\right.$$

$\widehat{{c}_{1}}$ is the crop with the highest probability among bell peppers, potatoes, and tomatoes in step 1, $T{h}_{1}$ is the average of the probability of true positive test samples in step 1.

Next, indicator function ${I}_{2}$ of step2, for evaluation sample $m$ ($m=1, \cdots , M, M=the\,number\,of\,evaluation\,samples\,not\,classified\,as{}^{^{\prime}}unknow{n}^{^{\prime}}in\,step1$)

$${I}_{2m}= \left\{\begin{array}{c}unknown\,\,\,\,\,\,\,\,\,\,\,if\,{p}_{m} < T{h}_{2}\\ \widehat{{c}_{2}}\,\,\,\,\,\,\,\,\,\,\,\,\,\,\,\,\,\,\,\,\,\,\,\,\,\,\,\,\,\,if\,{p}_{m} \ge T{h}_{2},\end{array}\right.$$

$\widehat{{c}_{2}}$ is the presence or absence of disease predicted in step 2 of the correctly predicted in step 1, $T{h}_{2}$ is the average of the probability of true positive test samples in step 2.

Finally, indicator function ${I}_{3}$ of step3, for evaluation sample $n$ ($n=1, \cdots , N, N=the\,number\,of\,evaluation\,samples\,not\,classified\,as{}^{^{\prime}}unknow{n}^{^{\prime}}in\,step1\,and\,2$)

$${I}_{3n}= \left\{\begin{array}{c}unknown\,\,\,\,\,\,\,\,\,\,\,\,\,\,if\,{p}_{n} < T{h}_{3}\\ \widehat{{c}_{3}}\,\,\,\,\,\,\,\,\,\,\,\,\,\,\,\,\,\,\,\,\,\,\,\,\,\,\,\,\,\,\,\,if\,{p}_{n} \ge T{h}_{3},\end{array}\right.$$

$\widehat{{c}_{3}}$ is a disease type if it is a result that there is a disease in potato or tomato through steps 1 and 2, $T{h}_{3}$ is the average of the probability of true positive test samples in step 3.

Two groups of image data were used to evaluate the performance of this model. The first was crop images used for model construction (model crops), and the second was crop images that were not used for model construction (non-model crops). Then, the results were distinguished by involvement of crops for model construction. In the case of detecting disease using non-model crops, ‘unknown’ was defined as correct, and assigned to model crops was defined as incorrect. In case of model crops, correct was accurate detection of disease, and incorrect was inaccurate detection of disease.

### Validation using lesion cropped image

A disease classification model was developed using cropped images of disease lesions. To investigate the performance of the disease classification model, whether it can be classified even with part of lesion images, the same diseased images from different crops were selected for validation. Thus, disease lesion images of early blight and late blight in potato and tomato were cropped and used to develop the classification model.

### Measurement of model performance

Accuracy, precision, recall, and F1-score were calculated to evaluate the performance of the classification model. They were calculated based on the confusion matrix. After creating a confusion matrix by comparing the model test result with the actual condition, true positive (TP), true negative (TN), false positive (FP), and false negative (FN) values are obtained. ‘True’ means the actual test sample was accurately predicted. Conversely, ‘false’ indicates a case of erroneous prediction. TP is when a test sample that is actually positive is accurately predicted as positive, and TN means that a negative test sample is accurately predicted as a negative sample. FP is a case in which the prediction result is positive when the sample is actually negative. FN refers to the number of cases in which the prediction result is negative, but the sample is actually positive. Accuracy refers to the percentage of correct predictions among all test results. Precision refers to the ratio of the actual positives among those predicted as positives. Conversely, recall refers to the percentage of actual positive samples predicted to be positive as a result of the test. The F1-score is the harmonic mean of precision and recall and is a corrected value to prevent misjudgment due to sample bias.

## Supplementary Information

Supplementary Information.

## Data Availability

Data used in the article are available in their original source. The PlantVillage dataset is available at https://github.com/spMohanty/PlantVillage-Dataset and the AI-hub dataset at https://aihub.or.kr/aihubdata/data/view.do?currMenu=115&topMenu=100&aihubDataSe=realm&dataSetSn=237.
