# Supplementary material for: Construction of deep learning-based disease detection model in plants
Source: Sci Rep. 2023 May 5;13:7331. doi: 10.1038/s41598-023-34549-2 (PMC10163233; doi:10.1038/s41598-023-34549-2)
Supplement: Supplementary file 1 — Supplementary Information. [file 41598_2023_34549_MOESM1_ESM.docx]

## Supplementary Material

# **Construction of Deep Learning-Based Disease Detection Model in Plants**

Minah Jung^1,2†^, Jong Seob Song^2†^, Ah-Young Shin^3,4†^, Beomjo Choi^3,5†^, Sangjin Go^3^, Suk-Yoon Kwon^3,6^, Juhan Park^2^, Sung Goo Park^1,7*^, and Yong-Min Kim^3,4,8*^

^1^Department of Functional Genomics, KRIBB School of Biological Science, Korea University of Science and Technology (UST), Daejeon, Republic of Korea

^2^Euclidsoft Co., Ltd, Daejeon, Republic of Korea

^3^Plant Systems Research Center, Korea Research Institute of Bioscience and Biotechnology (KRIBB), Daejeon, Republic of Korea

^4^Department of Bioinformatics, KRIBB School of Bioscience, Korea University of Science and Technology (UST), Daejeon, Republic of Korea

^5^Department of Environmental Horticulture, University of Seoul, Seoul, Republic of Korea

^6^Biosystems and Bioengineering Program, KRIBB School of Bioscience, Korea University of Science and Technology (UST), Daejeon, Korea

^7^Disease Target Structure Research Center, Korea Research Institute of Bioscience and Biotechnology (KRIBB), Daejeon, Republic of Korea

^8^Digital Bioinnovation Center, Korea Research Institute of Bioscience and Biotechnology (KRIBB), Daejeon, Republic of Korea

**Supplementary Fig. S1** Strawberry leaf images data for model evaluation.

**Supplementary Fig. S2** Excluded images.

**Supplementary Table S1** Architectures of five pre-trained CNN models.

**Supplementary Table S2** Hyperparameters for constructions of disease detection model.

**Supplementary Table S3** Performance measurement of five pre-trained CNN models using the test dataset.

**Supplementary Table S4** Disease symptoms of target diseases.

**Supplementary Table S5** Results of evaluation using other crops.

**Supplementary Table S6** Results of evaluation using bell pepper, potato, and tomato.

**Supplementary Table S7** Raw data for constructions of disease detection model.

**Supplementary Fig. S1** **Strawberry leaf images data for model evaluation.** (a) background removed images (PlantVillage) (b) field leaf images (AI-hub) (c) Results of evaluation using model 1 without strawberry and model 1 with strawberry.

**a**

**b**


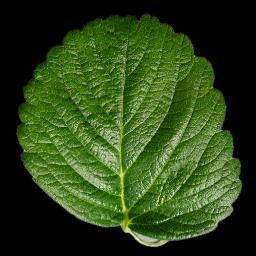

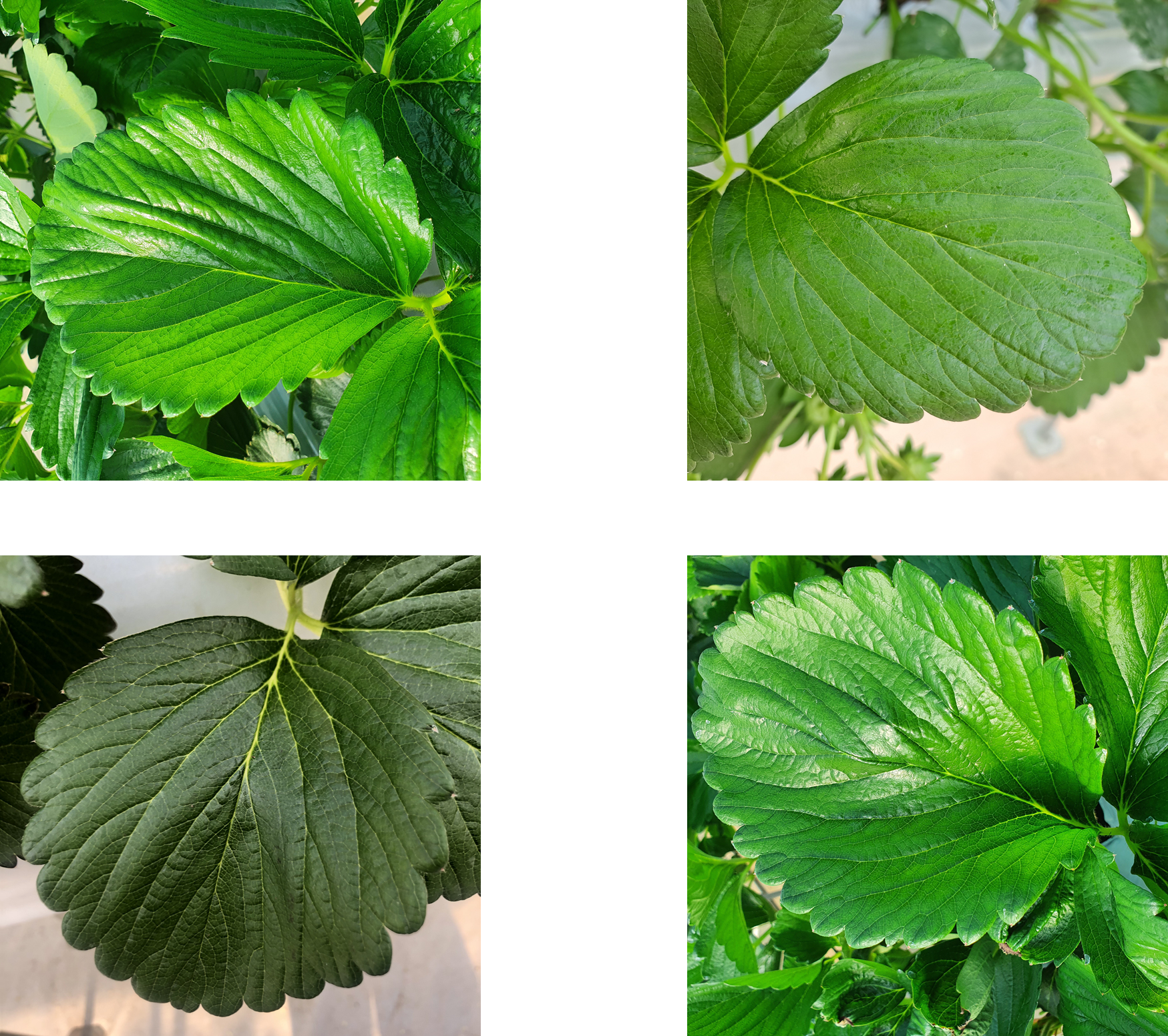

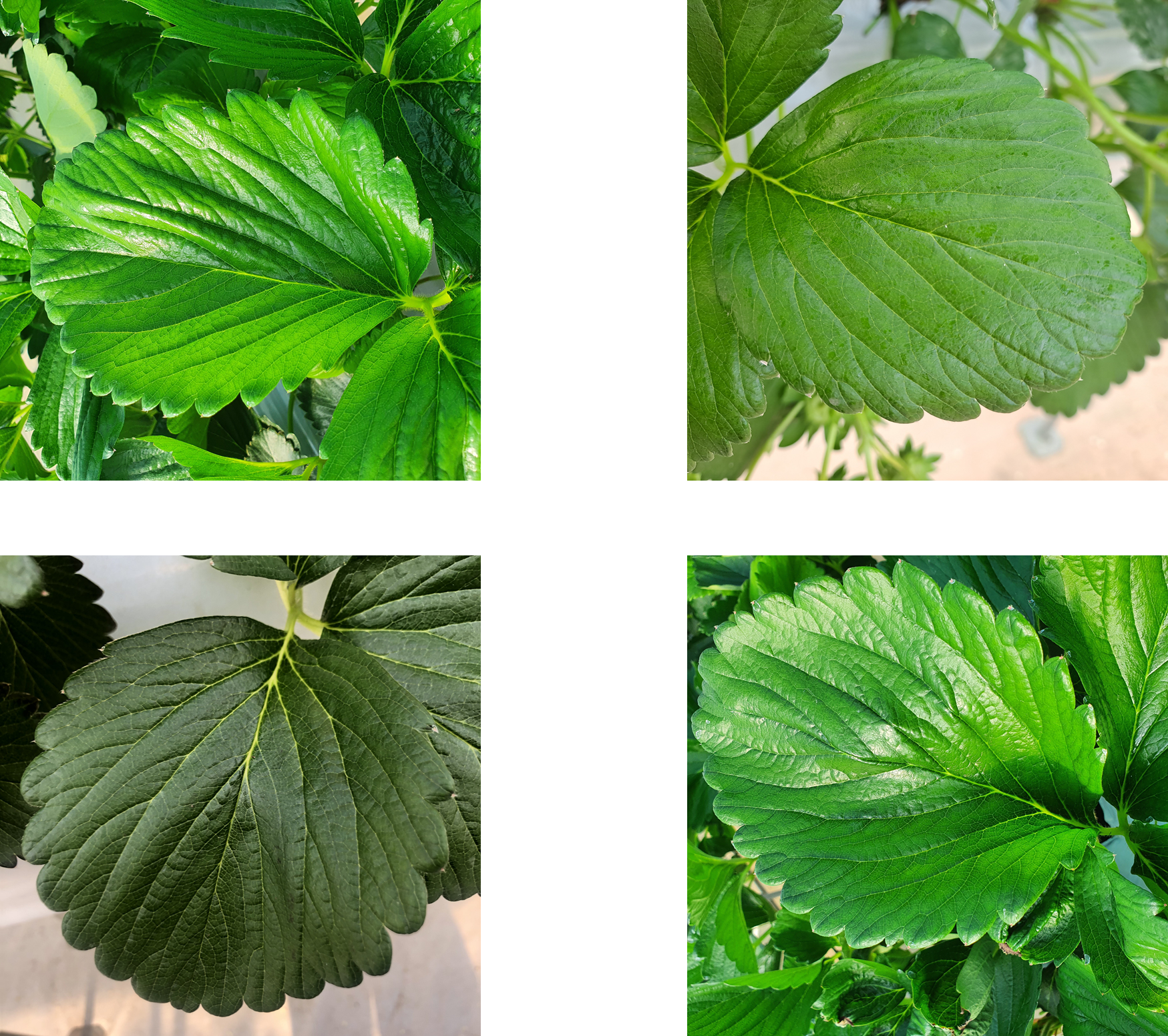

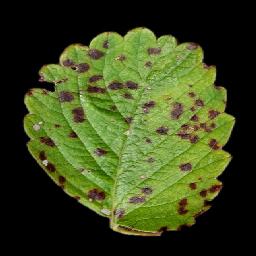


**c**


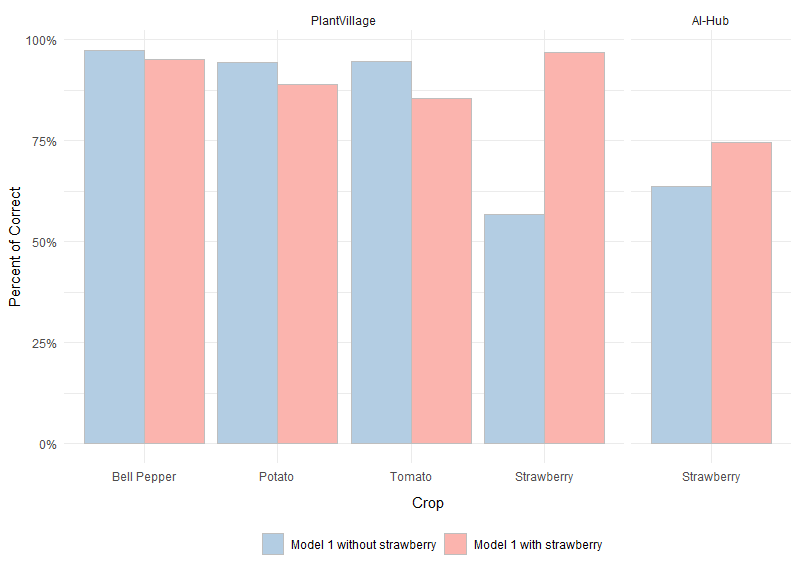


**
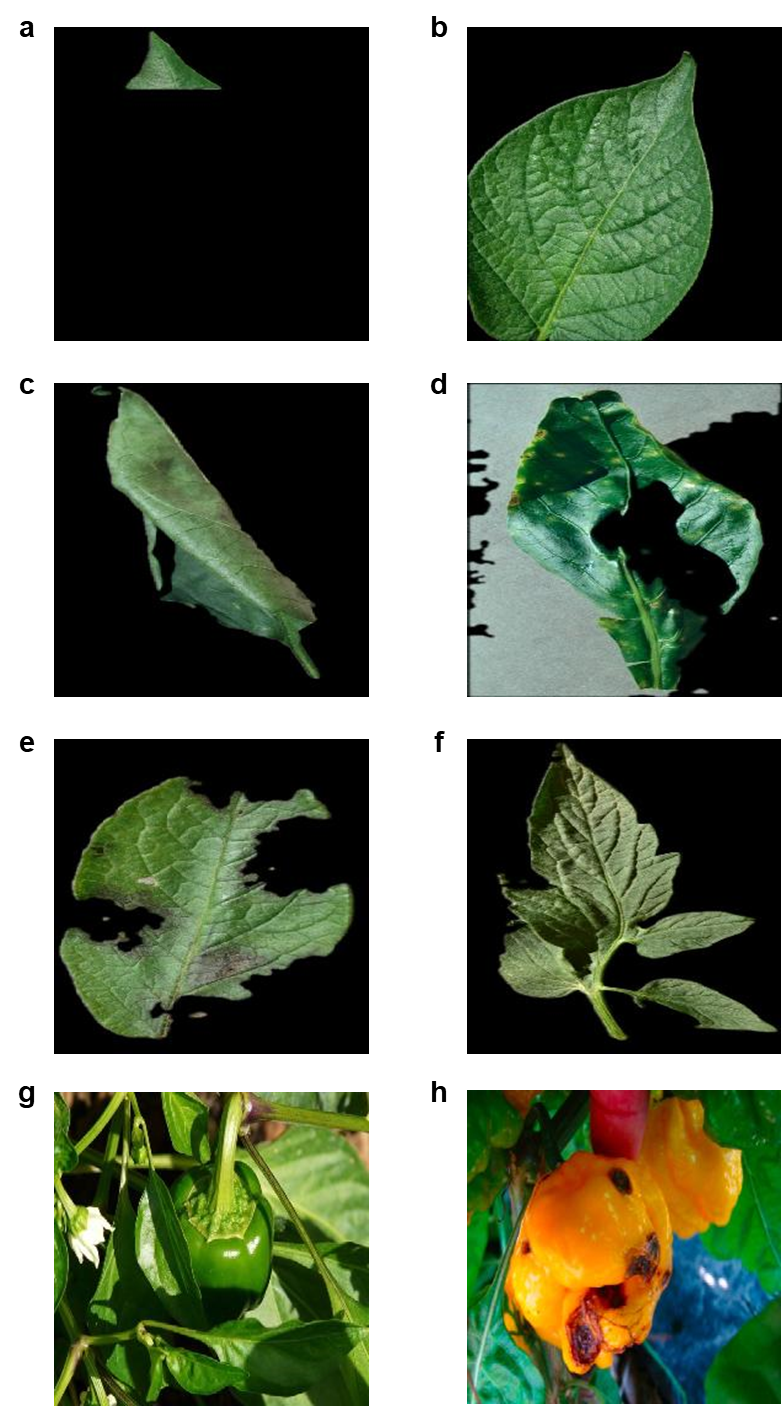
**

**Supplementary Fig. S2 Excluded images.** (a) partial image (cut off), (b) partial, (c) curled, (d) shade, (e) damage, (f) multiple leaves, (g) non-detectable and (h) fruit.

**Supplementary Table S1** Architectures of five pre-trained CNN models

| **ResNet50** | | **GoogLeNet** | | **VGG19** | | **EfficientNet** | |
| --- | --- | --- | --- | --- | --- | --- | --- |
| **Layer** | **Output** | **Layer** | **Output** | **Layer** | **Output** | **Layer** | **Output** |
| Input Conv1 MaxPooling Conv2 Conv3 Conv4 Conv5 AveragePooling FullyConnected Softmax | 224 × 224 × 3 112 × 112 × 64 56 × 56 × 64 56 × 56 × 256 28 × 28 × 512 14 × 14 × 1024 7 × 7 × 2048 1 × 1 × 2048 1 × 1 × 4 1 | Input Conv1_1 MaxPooling Conv2_1 Conv2_2 MaxPooling Inception3a Inception3b MaxPooling Inception4a Inception4b Inception4c Inception4d Inception4e MaxPooling Inception5a Inception5b AveragePooling DropOut Linear Softmax | 224 × 224 × 3 112 × 112 × 64 56 × 56 × 64 56 × 56 × 192 56 × 56 × 192 28 × 28 × 192 28 × 28 × 256 28 × 28 × 480 14 × 14 × 480 14 × 14 × 512 14 × 14 × 512 14 × 14 × 512 14 × 14 × 528 14 × 14 × 832 7 × 7 × 832 7 × 7 × 832 7 × 7 × 1024 1 × 1 × 1024 1 × 1 × 1024 1 × 1 × 1000 1 | Input Conv1_1 Conv1_2 MaxPooling Conv2_1 Conv2_2 MaxPooling Conv3_1 Conv3_2 Conv3_3 Conv3_4 MaxPooling Conv4_1 Conv4_2 Conv4_3 Conv4_3 MaxPooling FullyConnected1 FullyConnected2 FullyConnected3 Softmax | 224 × 224 × 3 224 × 224 × 64 224 × 224 × 64 112 × 112 × 128 112 × 112 × 128 112 × 112 × 128 56 × 56 × 256 56 × 56 × 256 56 × 56 × 256 56 × 56 × 256 56 × 56 × 256 28 × 28 × 512 28 × 28 × 512 28 × 28 × 512 28 × 28 × 512 28 × 28 × 512 7 × 7 × 512 1 × 1 × 4096 1 × 1 × 4096 1 × 1 × 1000 1 | Input Conv  MBConv1 MBConv6 MBConv6 MBConv6 MBConv6 MBConv6 MBConv6 MBConv6 MBConv6 MBConv6 MBConv6 MBConv6 MBConv6 MBConv6 MBConv6 MBConv6 Conv1_2 AveragePooling DropOut Linear Softmax | 224 × 224 × 3 112 × 112 × 32 112 × 112 × 16 56 × 56 × 24 56 × 56 × 24 28 × 28 × 40 28 × 28 × 40 28 × 28 × 80 28 × 28 × 80 28 × 28 × 80 14 × 14 × 112 14 × 14 × 112 14 × 14 × 112 7 × 7 × 192 7 × 7 × 192 7 × 7 × 192 7 × 7 × 192 7 × 7 × 320 7 × 7 × 1280 1 × 1 × 1280 1 × 1 × 1280 1 × 1 × 1000 1 |
| **AlexNet** | |  |  |  |  |  |  |
| **Layer** | **Output** |  |  |  |  |  |  |
| Input Conv1 MaxPooling Conv2 MaxPooling Conv3 Conv4 Conv5 MaxPooling FullyConnected1 FullyConnected2 FullyConnected3 Softmax | 224 × 224 × 3 55 × 55 × 96 27 × 27 × 96 27 × 27 × 256 13 × 13 × 256 13 × 13 × 384 13 × 13 × 384 13 × 13 × 256 6 × 6 × 256 1 × 1 × 4096 1 × 1 × 4096 1 × 1 × 1000 1 |  |  |  |  |  |  |

**Supplementary Table S2** Hyperparameters for constructions of disease detection model.

|  | **ResNet50** | **AlexNet** | **GoogLeNet** | **VGG19** | **EfficientNet** |
| --- | --- | --- | --- | --- | --- |
| Batch size | 2 | 2 | 2 | 2 | 2 |
| Activation | Softmax | Softmax | Softmax | Softmax | Softmax |
| Optimizer | ADAM | SGD | SGD | SGD | ADAM |
| Learning rate | 0.0001 | 0.0001 | 0.0001 | 0.0001 | 0.0001 |
| Epochs | 500 | 500 | 500 | 500 | 500 |
| Early stopping | Y | Y | Y | Y | Y |

**Supplementary Table S3** Performance measurement of five pre-trained CNN models using the test dataset.

| **Step** | | **Crop** | **Pre-trained Model** | **Performance** | | | |
| --- | --- | --- | --- | --- | --- | --- | --- |
|  |  |  |  | **Accuracy** | **Precision** | **Recall** | **F1-score** |
| **Ⅰ** | Crop Classification | All | ResNet50 | 91.84% | 92.86% | 91.84% | 91.66% |
|  |  |  | AlexNet | 96.87% | 96.87% | 96.87% | 96.86% |
|  |  |  | GoogLeNet | 99.08% | 99.08% | 99.08% | 99.08% |
|  |  |  | VGG19 | 98.71% | 98.71% | 98.71% | 98.71% |
|  |  |  | EfficientNet | 99.33% | 99.33% | 99.33% | 99.32% |
| **Ⅱ** | Disease Detection | Bell Pepper | ResNet50 | 98.32% | 95.90% | 100.00% | 97.91% |
|  |  |  | AlexNet | 99.16% | 99.46% | 98.40% | 98.93% |
|  |  |  | GoogLeNet | 100.00% | 100.00% | 100.00% | 100.00% |
|  |  |  | VGG19 | 99.58% | 99.47% | 99.47% | 99.47% |
|  |  |  | EfficientNet | 99.58% | 100.00% | 98.93% | 99.46% |
|  |  | Potato | ResNet50 | 99.45% | 100.00% | 99.40% | 99.70% |
|  |  |  | AlexNet | 98.90% | 99.70% | 99.10% | 99.40% |
|  |  |  | GoogLeNet | 99.45% | 99.70% | 99.70% | 99.70% |
|  |  |  | VGG19 | 100.00% | 100.00% | 100.00% | 100.00% |
|  |  |  | EfficientNet | 99.45% | 100.00% | 99.40% | 99.70% |
|  |  | Tomato | ResNet50 | 99.75% | 99.75% | 99.75% | 99.75% |
|  |  |  | AlexNet | 99.45% | 99.50% | 99.50% | 99.49% |
|  |  |  | GoogLeNet | 99.62% | 99.62% | 99.62% | 99.62% |
|  |  |  | VGG19 | 99.62% | 99.62% | 99.62% | 99.62% |
|  |  |  | EfficientNet | 98.23% | 98.33% | 98.23% | 98.24% |
| **Ⅲ** | Disease Classification | Potato | ResNet50 | 98.80% | 99.31% | 97.96% | 98.63% |
|  |  |  | AlexNet | 99.40% | 99.32% | 99.32% | 99.32% |
|  |  |  | GoogLeNet | 99.40% | 100.00% | 98.64% | 99.32% |
|  |  |  | VGG19 | 99.40% | 100.00% | 98.64% | 99.32% |
|  |  |  | EfficientNet | 99.40% | 100.00% | 98.64% | 99.32% |
|  |  | Tomato | ResNet50 | 87.80% | 90.31% | 87.80% | 88.39% |
|  |  |  | AlexNet | 95.45% | 95.32% | 95.45% | 95.35% |
|  |  |  | GoogLeNet | 95.81% | 96.29% | 95.81% | 95.82% |
|  |  |  | VGG19 | 95.08% | 94.92% | 95.08% | 94.96% |
|  |  |  | EfficientNet | 97.09% | 97.19% | 97.09% | 97.12% |

**Supplementary Table S4** Disease symptoms of target diseases.

| **Species** | **Diseases** | **Symptoms** | **References** |
| --- | --- | --- | --- |
| Bell Pepper | Bacterial  Spot | Symptoms begin as small, yellow-green lesions on young leaves which usually appear deformed and twisted, or as dark, water soaked, greasy-appearing lesions on older foliage. | Ritchie, D.F. 2000. Bacterial spot of pepper and tomato. The Plant Health  Instructor. DOI: 10.1094/PHI-I-2000-1027-01 |
|  |  | Lesions develop rapidly to a size of 0.25 to 0.5 cm (0.1 to 0.2 in.) wide and become tan to brownish-red. |  |
|  |  | Lesion shape is defined by leaf veinlets, so the shape is angular rather than the round shape. |  |
| Potato | Early  Blight | Initial symptoms on leaves appear as small 1-2 mm black or brown lesions and under conducive environmental conditions the lesions will enlarge and are often surrounded by a yellow halo. | Van der Waals, J. E., L. Korsten, and T. A. S. Aveling. "A review of early  blight of potato." African Plant Protection 7.2 (2001): 91-102. |
|  |  | Lesions greater than 10 mm in diameter often have dark pigmented concentric rings. | Kemmitt, G. 2002. Early blight of potato and tomato. The Plant Health  Instructor. DOI: 10.1094/PHI-I-2002-0809-01 |
|  | Late  Blight | They consist of small, pale to dark green spots the, change into brown or black lesions. | Henfling, Jan W. Late blight of potato. Vol. 4. International Potato Center, 1987. |
|  |  | A pale green or yellow border, a few millimeters wide, often separates dead from healthy tissue. | Schumann, G.L. and C. J. D’Arcy. 2000. Late blight of potato and tomato.  The Plant Health Instructor. DOI: 10.1094/PHI-I-2000-0724-01. Updated 2018 |
|  |  | Sporulation may be visible at the lower surface of the leaves as a white mildew surrounding the lesions. | No data was available |
| Tomato | Bacterial  Spot | Spots that appear on leaves and stems are small (up to 1 ⁄8 inch across), circular to irregular in shape, and have a slightly greasy feel. | Gleason, Mark Lawrence, and Brooke A. Edmunds. Tomato diseases and  disorders. Ames, IA: Iowa State University, University Extension, 2005. |
|  |  | As lesions enlarge, they often become surrounded by a yellow halo. |  |
|  | Early  Blight | Brown to black spots, 1 ⁄4 to 1 ⁄2 inch in diameter with dark edges, appear on lower leaves. |  |
|  |  | Dark, concentric rings often appear in leaf spots, resulting in the “target” appearance suggested by the common name. |  |
|  |  | Leaves turn yellow and dry up when only a few spots are present. |  |
|  | Late  Blight | It first appears as water-soaked areas that enlarge rapidly, forming irregular, greenish black blotches. |  |
|  | Tomato  Mosaic  Virus | ToMV causes yellow mosaic symptoms on the leaves and tomato fruits. | Mohamed, E. F. "Interaction between some viruses which attack tomato  (Lycopersicon esculentum Mill.) plants and their effect on growth and yield of tomato plants." J Amer Sci 6 (2010): 311-320. |
|  |  | Light and darker green mosaic leaf mottle, sometimes with distortion of younger leaves; this is the most common reaction in summer in glasshouses. |  |

**Supplementary Table S5** Results of evaluation using other crops.

| **Crops** | **Step** | **No. of Input Data** | **No. of Output Data** | | | |
| --- | --- | --- | --- | --- | --- | --- |
|  |  |  | **Correct** | | **Incorrect** | |
| Apple | Step 1 | 1,898 | 1,460 | (76.92%) | 438 | (23.08%) |
|  | Step 2 | 438 | 118 | (26.94%) | 320 | (73.06%) |
|  | Step 3 | 320 | 79 | (24.69%) | 241 | (75.31%) |
|  | Total | 1,898 | 1,657 | (87.30%) | 241 | (12.70%) |
| Corn | Step 1 | 3,023 | 2,783 | (92.06%) | 240 | (7.94%) |
|  | Step 2 | 240 | 5 | (2.08%) | 235 | (97.92%) |
|  | Step 3 | 235 | 31 | (13.19%) | 204 | (86.81%) |
|  | Total | 3,023 | 2,819 | (93.25%) | 204 | (6.75%) |
| Cherry | Step 1 | 1,340 | 612 | (45.67%) | 728 | (54.33%) |
|  | Step 2 | 728 | 193 | (26.51%) | 535 | (73.49%) |
|  | Step 3 | 535 | 0 | (0.00%) | 535 | (100.00%) |
|  | Total | 1,340 | 805 | (60.07%) | 535 | (39.93%) |
| Grape | Step 1 | 3,160 | 1,104 | (34.94%) | 2,056 | (65.06%) |
|  | Step 2 | 2,056 | 145 | (7.05%) | 1,911 | (92.95%) |
|  | Step 3 | 1,911 | 1,615 | (84.51%) | 296 | (15.49%) |
|  | Total | 3,160 | 2,864 | (91.66%) | 296 | (9.37%) |
| Peach | Step 1 | 839 | 769 | (37.14%) | 70 | (8.34%) |
|  | Step 2 | 70 | 26 | (29.55%) | 44 | (62.86%) |
|  | Step 3 | 44 | 13 | (29.55%) | 31 | (70.45%) |
|  | Total | 839 | 808 | (96.31%) | 31 | (3.69%) |
| Strawberry | Step 1 | 1,079 | 612 | (56.72%) | 467 | (43.28%) |
|  | Step 2 | 467 | 57 | (12.21%) | 410 | (87.79%) |
|  | Step 3 | 410 | 22 | (5.37%) | 388 | (94.63%) |
|  | Total | 1,079 | 691 | (64.04%) | 388 | (35.96%) |

**Supplementary Table S6** Results of evaluation using bell pepper, potato, and tomato.

| **Crops** | **Step** | **No. of Input Data** | **No. of Output Data** | | | |
| --- | --- | --- | --- | --- | --- | --- |
|  |  |  | **Correct** | | **Incorrect** | |
| Bell Pepper | Step 1 | 2,389 | 2,325 | (97.32%) | 64 | (2.68%) |
|  | Step 2 | 2,325 | 2,274 | (97.81%) | 51 | (2.19%) |
|  | Step 3 | 2,274 | 2,274 | (100.00%) | 0 | (0.00%) |
|  | Total | 2,389 | 2,274 | (95.19%) | 115 | (4.81%) |
| Potato | Step 1 | 1,810 | 1,707 | (94.31%) | 103 | (5.69%) |
|  | Step 2 | 1,707 | 1,681 | (98.48%) | 26 | (1.52%) |
|  | Step 3 | 1,681 | 1,606 | (95.54%) | 75 | (4.46%) |
|  | Total | 1,810 | 1,606 | (88.73%) | 204 | (11.27%) |
| Tomato | Step 1 | 3,949 | 3,740 | (94.71%) | 209 | (5.29%) |
|  | Step 2 | 3,740 | 3,321 | (88.80%) | 419 | (11.20%) |
|  | Step 3 | 3,321 | 3,196 | (96.24%) | 125 | (3.76%) |
|  | Total | 3,949 | 3,196 | (80.93%) | 753 | (19.07%) |

**Supplementary Table S7** Raw data for constructions of disease detection model.

| **Crops** | **Disease Status** | **Numbers** | | |
| --- | --- | --- | --- | --- |
|  |  | **Raw** | **Excluded** | **Used** |
| Apple | Healthy | 1,645 | 623 | 1,022 |
|  | Apple scab | 630 | 440 | 190 |
|  | Black rot | 621 | 126 | 495 |
|  | Cedar apple rust | 275 | 84 | 191 |
| Cherry | Healthy | 854 | 63 | 791 |
|  | Powdery mildew | 1,052 | 503 | 549 |
| Corn | Healthy | 1,162 | 272 | 890 |
|  | Cercospora leaf spot | 513 | 240 | 273 |
|  | Common rust | 1,192 | 57 | 1,135 |
|  | Northern leaf blight | 985 | 260 | 725 |
| Grape | Healthy | 423 | 99 | 324 |
|  | Black rot | 1,180 | 192 | 988 |
|  | Esca (Black Measles) | 1,384 | 341 | 1,043 |
|  | Leaf blight | 1,076 | 271 | 805 |
| Peach | Healthy | 360 | 178 | 182 |
|  | Bacterial spot | 2,297 | 1,640 | 657 |
| Bell Pepper | Healthy | 1,478 | 22 | 1,456 |
|  | Bacterial spot | 993 | 60 | 933 |
| Potato | Healthy | 152 | 4 | 148 |
|  | Early blight | 1,000 | 71 | 929 |
|  | Late blight | 1,001 | 268 | 733 |
| Strawberry | Healthy | 456 | 5 | 451 |
|  | Leaf scorch | 1,109 | 481 | 628 |
| Tomato | Healthy | 1,591 | 374 | 1,217 |
|  | Bacterial spot | 2,135 | 465 | 1,670 |
|  | Early blight | 992 | 690 | 302 |
|  | Late blight | 1,909 | 1,442 | 467 |
|  | Leaf mold | 674 | 62 | 612 |
|  | Septoria leaf spot | 1,231 | 190 | 1,041 |
|  | Spider mites | 1,676 | 322 | 1,354 |
|  | Target spot | 1,406 | 666 | 740 |
|  | Tomato mosaic virus | 373 | 92 | 281 |
|  | Tomato yellow leaf curl virus | 5,357 | 3,653 | 1,704 |
| Subtotal | Healthy | 8,121 | 1,640 | 6,481 |
|  | Disease | 31,061 | 12,616 | 18,445 |
| Total |  | 39,182 | 14,256 | 24,926 |
